# Detection of *AdeAB*, *TetA*, and *TetB* efflux pump genes in clinical isolates of tetracycline-resistant *Acinetobacter baumannii* from patients of Suez Canal University Hospitals

**DOI:** 10.1186/s12866-024-03735-1

**Published:** 2025-02-04

**Authors:** Hasnaa Azab, Aya Mohamed Askar, Noha M. Abd El-Fadeal, Amira A. A. Othman, Amal H. Rayan, Sally Khattab

**Affiliations:** 1 Microbiology and Immunology Department, Faculty of Medicine, Suez Canal University, Ismailia, Egypt; 2 Clinical and Chemical Pathology Department, Faculty of Medicine, Suez Canal University, Ismailia, Egypt; 3 Medical Biochemistry and Molecular Biology Department, Faculty of Medicine, Suez Canal University, Ismailia, Egypt; 4 Biochemistry Department, Ibn Sina National College for Medical Studies, Kingdom of Saudi Arabia, Jeddah, 22421 Saudi Arabia; 5 Oncology Diagnostic Unit, Faculty of Medicine, Suez Canal University, Ismailia, Egypt; 6 Internal Medicine Department, Faculty of Medicine, Suez University, Suez, 43511 Egypt; 7 Department of Basic Medical Science, College of Medicine, AlMaarefa University, Riyadh, Saudi Arabia; 8 Faculty of Medicine, Suez Canal University, Ismailia, Egypt

**Keywords:** *A. Baumannii*, Efflux pump inhibitor, Tetracycline, Multi-drug resistance

## Abstract

**Background:**

*Acinetobacter baumannii* is an opportunistic bacteria associated primarily with hospital-acquired infections. Its tendency to acquire or donate resistance genes to neighboring bacteria is a major concern. Tetracyclines have shown promise in treating *A. baumannii* infections, but tetracycline resistance is growing globally in *A. baumannii* isolates.

**Objectives:**

The study aimed to study (1) the prevalence of multidrug-resistant (MDR) *A. baumannii* infections at Suez Canal University Hospitals, (2) the distribution of efflux pump genes *AdeA &B*, *TetA*, and *TetB*, and (3) the effect of efflux pump inhibitor (CCCP) on tetracycline-resistant isolates.

**Methods:**

Clinical samples (457) were collected (blood, urine, sputum, ETA, pus, and pleural fluid), followed by *A. baumannii* isolation and identification, PCR detection of efflux pump genes, and detection of tetracycline susceptibility and its MIC before and after treatment with the efflux pump inhibitor (CCCP).

**Results:**

A total of 31 *A. baumannii* isolates were recovered (6.78%). The highest rate of isolation was from the ICU (48.3%) from the ET aspirate samples (48.3%). The efflux system AdeA and TetB genes were distributed in 100% of isolates, whereas AdeB was found in 93.5% of isolates and the TetA gene in 87.1% of isolates. All *A. baumannii* isolates were MDR showing resistance to three or more classes of antibiotics. 45% of the isolates showed a 4-fold reduction of MIC and 12.9% showed a 2-fold reduction in the MIC.

**Conclusions:**

Efflux pump is an important mechanism for tetracycline resistance among *A. baumannii* isolates.

## Introduction

*Acinetobacter baumannii* (*A. baumannii*), an aerobic, pleomorphic, non-motile, gram-negative, coccobacilli, is an opportunistic bacterial pathogen primarily associated with hospital-acquired infections (nosocomial), particularly among immunocompromised individuals causing bacteremia, urinary tract infection, ventilator-associated pneumonia (VAP), and wound infections. Sporadic cases of peritonitis, endocarditis, meningitis, osteomyelitis, and arthritis have also been reported [1]. VAP is the most frequent ICU-acquired infection, occurring in 9 to 24% of patients intubated for longer than 48 h. Colonization of the digestive tract in intensive care unit patients is an important epidemiologic reservoir for multi-drug-resistant *A. baumannii* infections in hospital outbreaks [2]. *A. baumannii* naturally encodes efflux pumps, providing it with intrinsic resistance to antibiotics beside low membrane permeability, both of which allow it to survive a variety of antibiotics used, making it challenging to treat in some clinical settings, particularly with critically ill patients. Thus, there are now just a few antibiotics projected to be effective against the severe types of *A. baumannii* infections leading to the augmentation of fatality rates and healthcare expenditures [3].

Unfortunately, several methods, including an efflux pump, a ribosome protection system, and enzyme modification, can lead to bacterial antibiotic resistance. *A. baumannii*-resistant strains continue to appear and spread, and there aren’t many effective treatment choices [4]. According to an Egyptian study, 61.4% of the *Acinetobacter spp.* isolates were found to be multi- and extensive drug-resistant bacteria [5]. For this reason, alternative antibiotics have been studied for use in clinical settings. Tetracyclines, such as doxycycline and minocycline, have demonstrated encouraging microbiological and clinical efficacy in the treatment of *A. baumannii* infections. It has been found that tetracyclines can be successfully used in conjunction with other antibiotics to treat 87.5% of bloodstream infections and 71.9% of respiratory infections [6].

The efflux pumps are involved in clinically relevant resistance to antimicrobial agents. They are responsible for reducing antibiotic accumulation via transporting antibiotics outside the bacterial cells [7]. There are five families of efflux-pump proteins that are associated with multidrug resistance (MDR) in bacteria, based on amino acid sequence homology, (1) the “multidrug and toxic compound extrusion” family (MATE), (2) the “adenosine triphosphate (ATP)-binding cassette” superfamily (ABC), (3) the “small multidrug resistance” family (SMR), (4) the “major facilitator superfamily (MFS), and (5) the “resistance-nodulation-cell division” family (RND). Drug efflux pumps are found in both gram-negative and gram-positive bacteria, with more complexity in Gram-negative ones due to the molecular architecture of the cell envelope (reduced drug intake) and efflux pumps (active drug export). *A. baumannii* antimicrobial resistance has been linked to more than one class of efflux pumps: the SMR family, the MATE family, and the RND family [8]. The tetracycline-specific efflux pump system in *A. baumannii* is the synergistic act of TetA and TetB, members of the MFS superfamily efflux pump, and AdeABC, members of the RND superfamily efflux pump. TetA efflux provides resistance against tetracycline, but not against minocycline or doxycycline, while TetB efflux provides resistance against tigecycline, but not against tetracycline and minocycline. Additionally, it has been discovered that TetA functions in concert with AdeABC to provide a crucial tigecycline resistance mechanism [9].

Since efflux pumps have a significant role in the resistance mechanisms of *A. baumannii*, efflux pump inhibitors (EPIs) have been extensively investigated to confer one potential solution to combat antibiotic resistance by blocking their efflux pumps via “competitive or non-competitive inhibition”, “dissipation” of the proton gradient needed by efflux pumps to export antibiotics, “suppression” of the expression of genes that encode efflux pumps, “blocking the inner or outer membrane protein” used for efflux, “disruption” of the efflux pump assembly, and “changing the structure of the medication” so that it cannot be recognized [10]. One of these inhibitors is carbonyl cyanide 3-chlorophenylhydrazone (CCCP), an oxidative phosphorylation uncoupler that affects bacterial membrane ionic gradients. This compound has been efficiently utilized in combination with antibiotics to increase their susceptibility [11].

Given the high prevalence of multidrug-resistant (MDR) *A. baumannii* infections at Suez Canal University Hospitals, this study aimed to investigate the distribution of *AdeAB*, *TetA*, and *TetB* genes and the effect of efflux pump inhibitor (CCCP) on tetracycline-resistant isolates among isolates from patients in this institution.

## Materials and methods

### Study population and design

This observational cross-sectional descriptive study was conducted at the Suez Canal University Hospitals in Ismailia, Egypt, from September 2022 to September 2023. Samples were collected from different intensive care units (ICUs) and other hospital wards and were processed in the Medical Microbiology and Immunology Department laboratory, Faculty of Medicine, Suez Canal University.

### Ethical considerations

The current study was implemented in coordination with the guidelines of the Declaration of Helsinki. Ethical approval was gained from the Research Ethics Committee of the Faculty of Medicine, Suez Canal University, Egypt, # 5024. Informed consent was obtained from the patients, which addressed all the steps of the study and their right to withdraw at any time.

### Inclusion criteria

Patients of both sexes (male & female) and all age groups who showed signs and symptoms of infections and agreed to participate were included in this study. Samples included were urine from catheterized and non-catheterized patients, respiratory specimens from intubated and non-intubated patients, Pus and wound swabs, pleural fluid, and blood.

### Exclusion criteria

Patients who received antibiotic treatment in the last 48 h or refused to participate were excluded.

## Study procedure

### Full history taking, and thorough physical examination

All enrolled patients were asked and examined for underlying medical conditions or certain types of healthcare exposures, such as:

- Immunocompromising conditions.
- Recent frequent or prolonged stays in health care settings.
- Invasive medical devices such as breathing tubes, feeding tubes, and central venous catheters.
- Open wounds.
- A history of taking certain antibiotics for long periods.

For every patient admitted to internal wards or intensive care units, the following data was found age, length of stay, number of days needed for mechanical ventilation, and survival until hospital discharge. Additionally, microbiological results were obtained to identify *A. baumannii-*positive cultures.

### Samples collection and preservation

Various samples were collected throughout the study for presumptive *A. baumannii* isolation and identification according to standard laboratory procedures, including culture on Enriched and selective agar media and incubation under appropriate conditions following the guidelines provided by the Clinical and Laboratory Standards Institute (CLSI) [12] and the World Health Organization (WHO) [13]. We followed the CDC’s recommendations to prevent the spread of drug-resistant Acinetobacter spp. These included implementing contact isolation precautions, enhancing environmental cleaning, using dedicated patient care equipment, and ensuring the prudent use of antibiotics. Additionally, healthcare personnel adhered to strict infection control practices, such as wearing gowns and gloves when entering patient rooms and maintaining rigorous hand hygiene protocols [14].

#### a. blood samples

Blood samples were taken before the onset of antimicrobial therapy and throughout the feverish phase when feasible. Ten milliliters of blood were extracted and promptly injected into sterile blood culture bottles holding fifty milliliters of brain heart infusion (BHI) broth after the skin had been cleaned and disinfected with 70% alcohol. Two milliliters of blood were taken from neonatal patients and placed into sterile culture flasks with ten milliliters of BHI broth. Culture flasks were brought straight to the lab and kept there between 35 and 37 °C.

#### b. urine samples

Urine samples were taken midstream in non-catheterized patients and placed in a sterile wide-mouthed container. In catheterized patients, their catheters had clamped off. After cleaning the needle puncture site and inserting the syringe at a 45° angle, urine was aspirated using a syringe. Urine specimens were rapidly transported to the laboratory (less than two hours post-voiding). Urine samples were refrigerated at 4 °C until processing if a delay was expected.

#### c. Sputum samples

Early morning samples were collected from conscious patients with lower respiratory tract infections. Patients were instructed to gargle with water immediately before collecting samples to reduce the number of contaminating oropharyngeal bacteria. Sputum was collected by asking patients to cough deeply into a sterile wide-mouthed container with a tightly fitted cap. Only thick sputum secretions from the lung were acceptable.

#### d. endotracheal aspirate samples (ETA)

A sterile silicon tracheal catheter was used to collect ETA. It was inserted through the trachea until resistance (the level of the trachea’s carina) was met, at this point it was retracted about 2 cm. The vacuum was then released, and the catheter was carefully removed using rotating motions. Following the catheter’s withdrawal, the exudates were flushed into a sterile container using a sterile syringe and 2 mL of sterile 0.9% normal saline. After that, the samples were delivered to the microbiology lab in a maximum of two hours.

#### e. pus and wound exudate samples

The traumatized region was cleaned with sterile saline to remove contaminated materials like slough, necrotic tissue, or dry exudates. Pus and wound exudates were either collected by inserting a sterile cotton swab deeply down the leading edge of the wound or aspirated using a syringe. The specimens were immediately brought to the lab in Amie’s transport medium.

#### f. pleural fluid specimens

The pleural fluid specimen was aseptically obtained using thoracocentesis and placed in a tube containing a sterile anticoagulant (ethylenediaminetetraacetic acid, or EDTA) before being sent right away to the lab.

### Bacterial isolation and identification

For bacterial isolation, all collected samples were inoculated into blood agar and MacConkey agar media (Oxoid, UK) and incubated aerobically at 35 ± 2˚C for 24 h. For initial identification of the isolates, they underwent a hanging drop test, and biochemical tests including “catalase, citrate, oxidase, coagulase, oxidative fermentation, indole, Methyl Red (MR), Voges-Proskauer (VP), urease, H_2_S production, gelatin hydrolysis, and bile solubility”, and growth at temperatures of 37 °C and 44 °C.

Molecular confirmation of *A. baumannii* isolates (genotypic characteristic) was performed using conventional PCR amplification of the “*Bla*_OXA−51−like_ gene”. DNA of the isolates was extracted using a DNA extraction kit according to the manufacturer’s instructions. DNA extraction kit was supplied from “QIAGEN Company (Cat. No.27200-4)”. DNA concentration and purity were measured using a spectrophotometer. The PCR mixture was done in a total volume of 25 µl including 1 µl MgCl_2_ (1.5 mM), 0.3 µl Taq DNA polymerase (500 U), 2.5 µl 10x PCR buffer, 0.5 µl dNTP (200 µM), 1 µl of each primer) 10 pmol/ml) and 2 µl of DNA template (5 ng genomic DNA). The primer sets “F: 5’-TAATGCTTTGATCGGCCTTG-3’ and R: 5’-TGGATTGCACTTCATCTTGG-3’” were used for amplification of the “OXA-51-like” gene [15]. The amplification conditions: initial denaturation at 94 °C for 5 min, and 30 cycles of 94 °C for 45 s, 52 °C for 40 s, 72 °C for 45 s, and a final extension at 72 °C for 6 min. Amplified fragments were separated by electrophoresis in 2% agarose gel at 5 volts/cm for 2 h. Finally, the fragments of the *Bla*OXA-51-like gene with 353 bp size were stained with ethidium bromide and detected under a UV transilluminator documentation system [16].

### Molecular detection of Efflux pump genes (AdeA, AdeB, TetA, and TetB)

Multi-drug-resistant *A. baumannii* isolates were screened for four Efflux pump genes (*AdeA*,* AdeB*,* TetA*, and *TetB*). They were tested by conventional PCR using the primers illustrated in Table 1. PCR cycling conditions included initial denaturation at 94 °C for 5 min, and 30 cycles of 94 °C for 45 s, 56–58 °C for 40 s, 72 °C for 45 s, and a final extension at 72 °C for 6 min. Amplified fragments were separated by electrophoresis in 2% agarose gel at 5 volts/cm for 2 h.

**Table 1:** Gene-specific primers used to detect efflux pump genes in *A. Baumannii* isolates

Target gene	Primer Sequence (5` to 3`)		Amplicon size (bp)	Melting Temp.	CG content	Annealing temp.		Ref.	
*AdeA*	**F** **R**	TGCCCAATACGCCCAGAAAT GTCAGGCTCTAGCCGATGTC	106	60.03 59.97	50% 60%		58 °C		[24]
*AdeB*	**F** **R**	AACGTCGACCTGAGCCATTT GTGCGACTTATCCTGGTGCT	185	59.97 60.11	50% 55%		58 °C		[25]
*TetA*	**F** **R**	GCTACATCCTGCTTGCCTTC CATAGATCGCCGTGAAGAGG	210	58.98 57.94	55% 55%		56 °C		[26]
*TetB*	**F** **R**	GTAAAGCGATCCCACCACCA ACCACCTCAGCTTCTCAACG	586	60.04 59.97	55% 55%		58 °C		[27]

### Antimicrobial sensitivity testing

#### a. standard disk diffusion method

Phenotypic detection of antibiotic resistance was done using the “Kirby-Bauer disk diffusion method” on Mueller Hinton agar and incubated at 35ºC for 16–18 h according to Clinical and Laboratory Standard Institute guidelines of CLSI, 2022, using *A. baumannii* ATCC19606 as a reference strain [17]. *A. baumannii isolate* ATCC 19,606 was recovered in the US before 1948. It has been used as a reference and model organism in many studies involving antibiotic resistance and pathogenesis of *A. baumannii*. The *Acinetobacter baumannii* ATCC 19,606 strain is known to harbor efflux pump genes such as *TetA* and *AdeABC*, which mediate resistance to tetracycline. The expression of the *AdeABC* efflux pump genes is tightly regulated by the AdeRS two-component system in *A. baumannii* ATCC 19,606. Both amino acid substitutions and transposon insertion have been shown to continuously turn on the AdeRS two-component system and then constitutively activate *AdeABC* efflux pump gene expression in clinical isolates or laboratory mutants [18]. The MIC of tetracycline for this strain, with a value of 6 µg/ml to tetracyclines [19]. Inhibition of *A. baumannii* efflux pump genes by CCCP inhibitor restores its antimicrobial susceptibility [20].

The following antibiotic disks were used: “Cefepime (30µg), Ceftazidime (30µg), Meropenem (10µg), Gentamycin (10µg), Amikacin (30µg), Ciprofloxacin (5µg), Levofloxacin (5µg), Tetracycline (30µg), and Doxycycline (30µg)”. Bacterial isolates that are resistant to at least one agent in at least three classes of antibiotics are classified as multidrug-resistant (MDR), while isolates that are responsive to just one or two antimicrobial classes are classified as extensively drug-resistant (XDR) [21].

#### b. minimal inhibitory concentration determination

The agar dilution method was used to assess the minimal inhibitory concentration (MIC) of tetracycline both before and after treatment with the efflux pump inhibitor “carbonylcyanide 3-chlorophenylhydrazone” (CCCP) (Sigma-Aldrich, Dorset, United Kingdom) [22].

CCCP was added to Mueller-Hinton (M-H) agar plates, leading to increased intracellular concentration of the antibiotic reducing the MIC in isolates with active efflux pumps. M-H agar plates were prepared with 0.5 to 1024 µg/mL concentration of tetracycline and CCCP (25 µg/mL) was added to each. Then, the MIC of tetracycline was determined for all tetracycline-resistant isolates against the *A. baumannii* isolate. M-H agar plates with CCCP without the antibiotic were used as controls. The effect of the efflux pump inhibitor was determined by detecting a ≥ 4-fold increase in the susceptibility after treatment with CCCP [23].

### **Statistical analysis**

Data collected will be reviewed, coded, and statistically analyzed using Statistical Package for the Social Science (SPSS) program version 28 (Inc, Chicago, Illinois, USA). Data presentation was performed via tables and graphs. Qualitative data were presented as numbers and percentages while quantitative data were presented as mean ± Standard Deviation. Fisher’s exact tests were used for qualitative variables. The McNemar test was used to compare tetracycline resistance pre- versus post-adding CCCP. Also, the Wilcoxon signed-rank test was used to compare MIC scores. A *p-*value of *< 0.05* was considered statistically significant.

## Results

### Isolation, identification, and phenotypic detection of A. Baumannii isolates

A total of 457 examined clinical samples were analyzed for bacterial isolation. A total of 31 *A. baumannii* isolates were recovered (6.78%). Other organisms represented 426 samples (93.3%) of the isolated microorganisms.

The ages of the patients ranged from 13 days old to 72 years old. *A. baumannii* isolates were obtained more frequently from males than females (Table 2**).**

**Table 2:** Frequency distribution of patients infected with *A. Baumannii* according to gender and age (*N* = 31)

Demographic data	
**Gender**	**Male n.(%)** 17 (54.8%
	**Female n.(%)** 14 (45.2%)
**Age**	**Range** 13 days − 72 years
	**Mean ± SD** 46 ± 17.6

The highest rate of *A. baumannii* isolation was from the ICU (48.3%), while the lowest rate was from the urology and orthopedics departments (3.3%, each) (Table 3). The highest rate of *A. baumannii* isolation was from the ET aspirate samples (48.3%), while the lowest rate was from wound swabs (3.2%) (Table 4).

**Table 3:** Frequency distribution of the isolated MDR *A. Baumannii* isolates according to the hospital wards from which the sample was obtained (*N* = 31)

Hospital Section	Frequency	Percentage %
ICU	15	48.3%
Internal Medicine Wards	5	16.2%
Surgery	3	9.7%
NICU	2	6.4%
Burn Unit	2	6.4%
PICU	2	6.4%
Urology	1	3.3%
Orthopedics	1	3.3%
Total	31	100%

**ICU**: Intensive Care Unit, **NICU**: Neonatal Intensive Care Unit, **PICU**: Pediatric Intensive Care Unit

**Table 4:** Frequency distribution of the isolated MDR *A. baumannii* strains according to the type of the samples (*N* = 31)

Type of specimen	Frequency	Percentage %
ET aspirate	15	48.3%
Sputum	3	10%
Urine	5	16.1%
Blood	3	10%
Pleural effusion	2	6.2%
Pus	2	6.2%
Wound Swab	1	3.2%
Total	31	100%

**ET aspirate**: Endotracheal aspirate

Preliminary isolation and identification of *Acinetobacter spp.* based on Gram-negative staining and colony morphology. *A. baumannii* are Gram -ve, non-motile coccobacilli. Macroscopic characterization of colonies on blood agar and MacConkey agar showed small smooth, opaque, raised, and creamy colonies on blood agar, and pure purple or mucoid colonies on MacConkey agar. Biochemical tests used to identify the *A. baumannii* isolates were reported in Table 5.

**Table 5:** Biochemical tests are used to identify *A. Baumannii* isolates

Basic Characteristic tests	Properties (A. baumannii)
Catalase, Citrate	Positive (+ ve)
Oxidase, Coagulase, Indole, MR, VP, Urease, H_2_S, Gelatin Hydrolysis, Bile Solubility	Negative (-ve)
Oxidative fermentation	Non- Fermentative (Oxidative)

### Genotypic detection of A. Baumannii isolates and efflux pumps genes

**Fig. 1:**
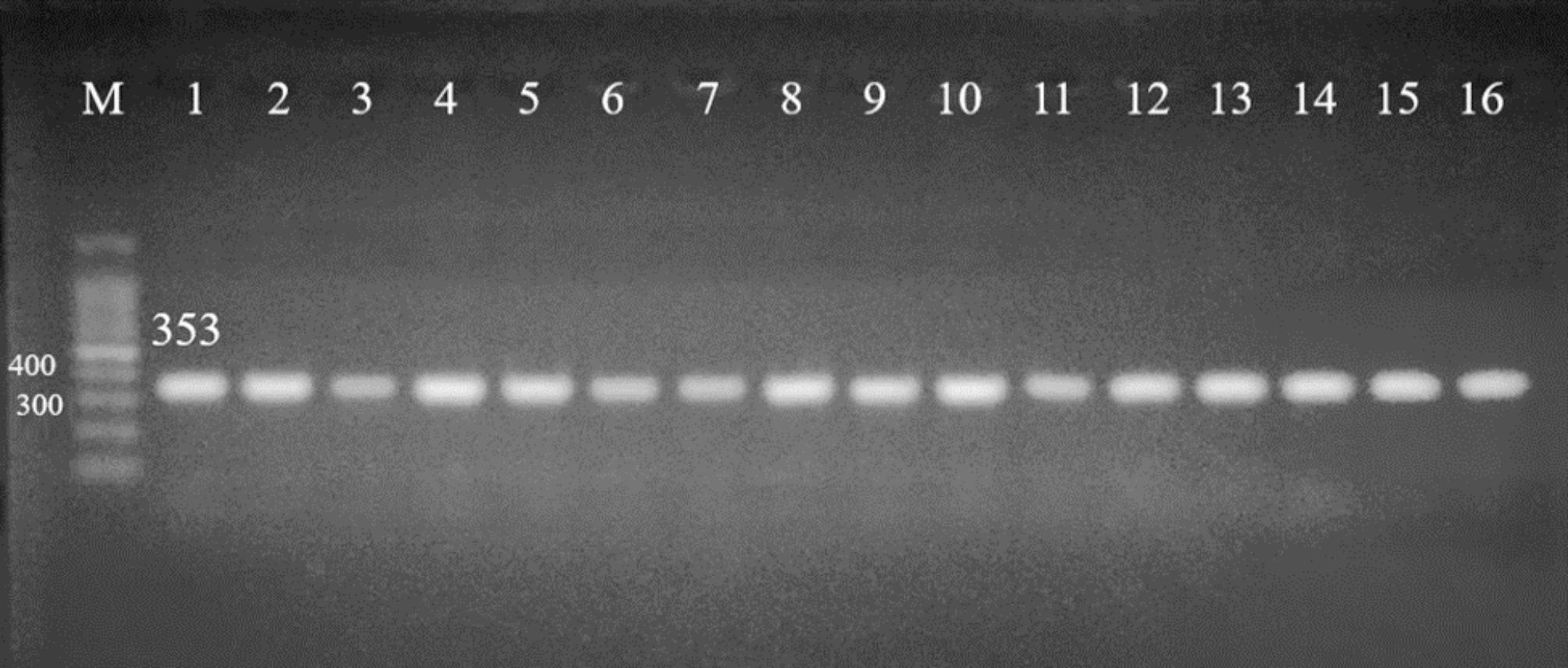
Detection of *Bla*_OXA−51−like_ gene by agarose gel electrophoresis. Lane M shows a 100 bp molecular strand DNA ladder. Lane 1–16 shows positive samples (353 bp)

**Fig. 2:**
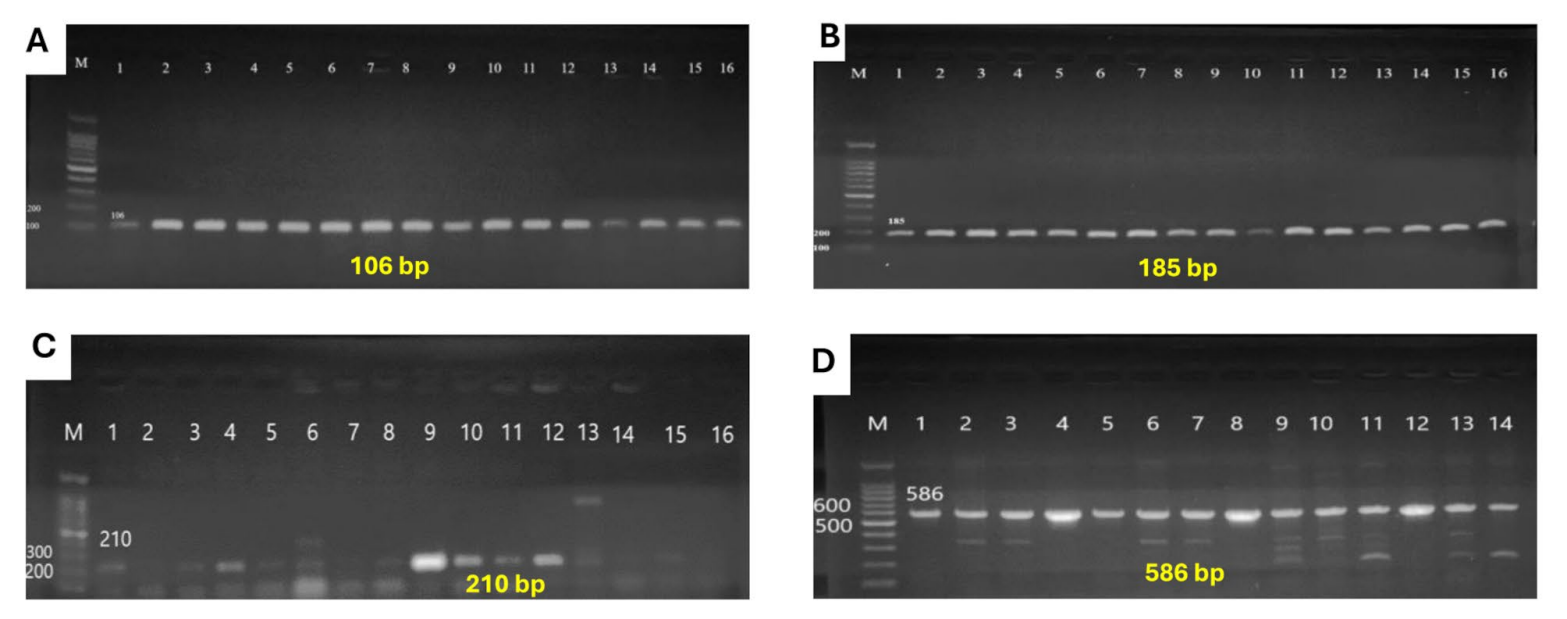
Detection of efflux pump genes by agarose gel electrophoresis. Lane M shows a 100 bp molecular strand DNA ladder, **A**: *AdeA* gene; Lane 1–16 shows positive samples (106 bp), **B**: *AdeB* gene; Lane 1–16 shows positive samples (185 bp), **C**: *TetA* gene; Lane 9, 10,11 and 12 show positive specimens (210 bp), D: *TetB* gene; Lanes 1–14 shows positive specimens (586 bp)

The 31 isolates (100%) were confirmed to be *A. baumannii* by conventional PCR detection of the *Bla*_OXA−51−like_ gene by gel electrophoresis at 353 bp (Fig. 1). *AdeA* and *TetB* genes were detected in 100% of isolates (31 isolates), where *AdeB* was detected in 93.5% of isolates (29 isolates) and *TetA* gene in 87.1% of isolates (27 isolates). Detection of the genes *AdeA*,* AdeB*,* TetA*, and *TetB* by gel electrophoresis were at 106 bp, 185 bp, 210 bp, and 586 bp respectively (Fig. 2A-D).

### Antibiotic susceptibility test

**Fig. 3:**
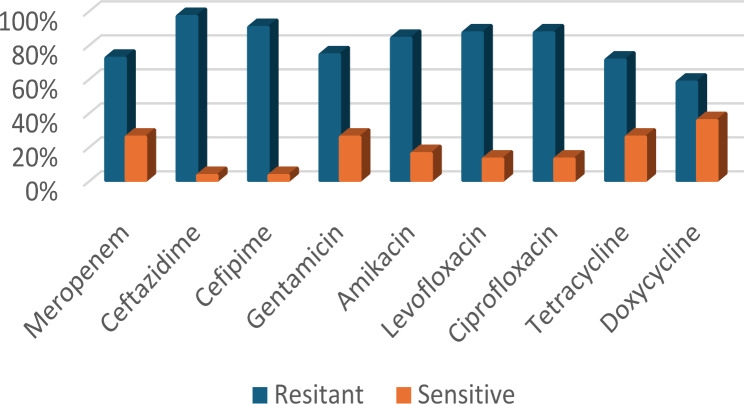
Antibiotic susceptibility pattern of the studied MDR *A. baumannii* isolates by the disc diffusion method. The figure shows the percentage of resistance to antibiotics

An antibiotic susceptibility test was done on the 31 *A. baumannii* isolates by using the disk diffusion method (Kirby Bauer method). It showed highest resistance to ceftazidime (30 isolates, 96.8%) followed by cefepime (28 isolates, 90.3%), levofloxacin and ciprofloxacin (27 isolates, 87.1%), amikacin (26 isolates, 83.9%), gentamicin and meropenem (23 isolates, 74.2%) and tetracycline (22 isolates, 71%). The least resistance was to doxycycline (18 isolates, 58.1%) (Fig. 3).

### The minimal inhibitory concentration of tetracycline

**Fig. 4:**
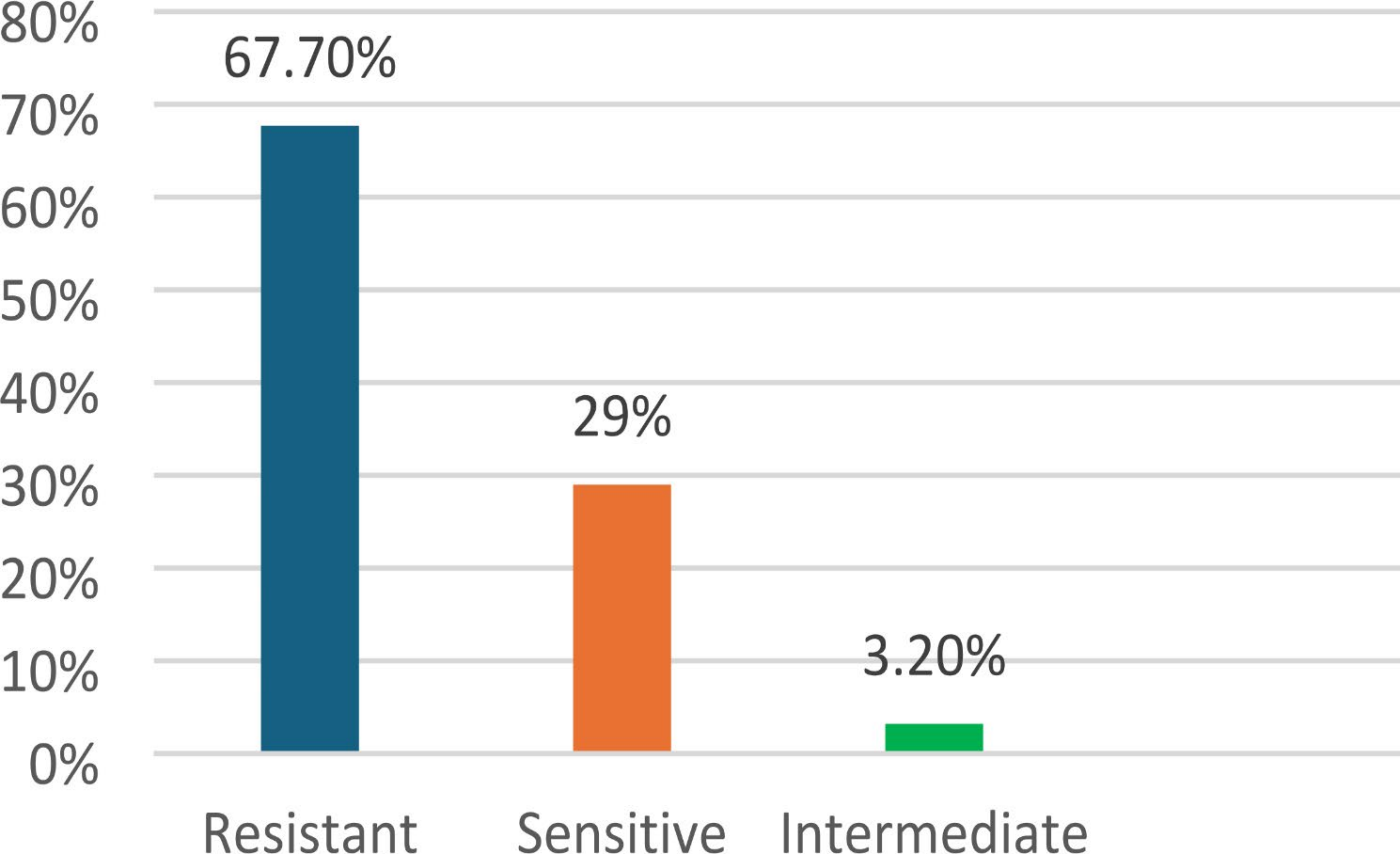
Distribution of tetracycline susceptibility by agar dilution method. The figure shows that 67.7% of isolates were resistant to tetracycline

**Fig. 5:**
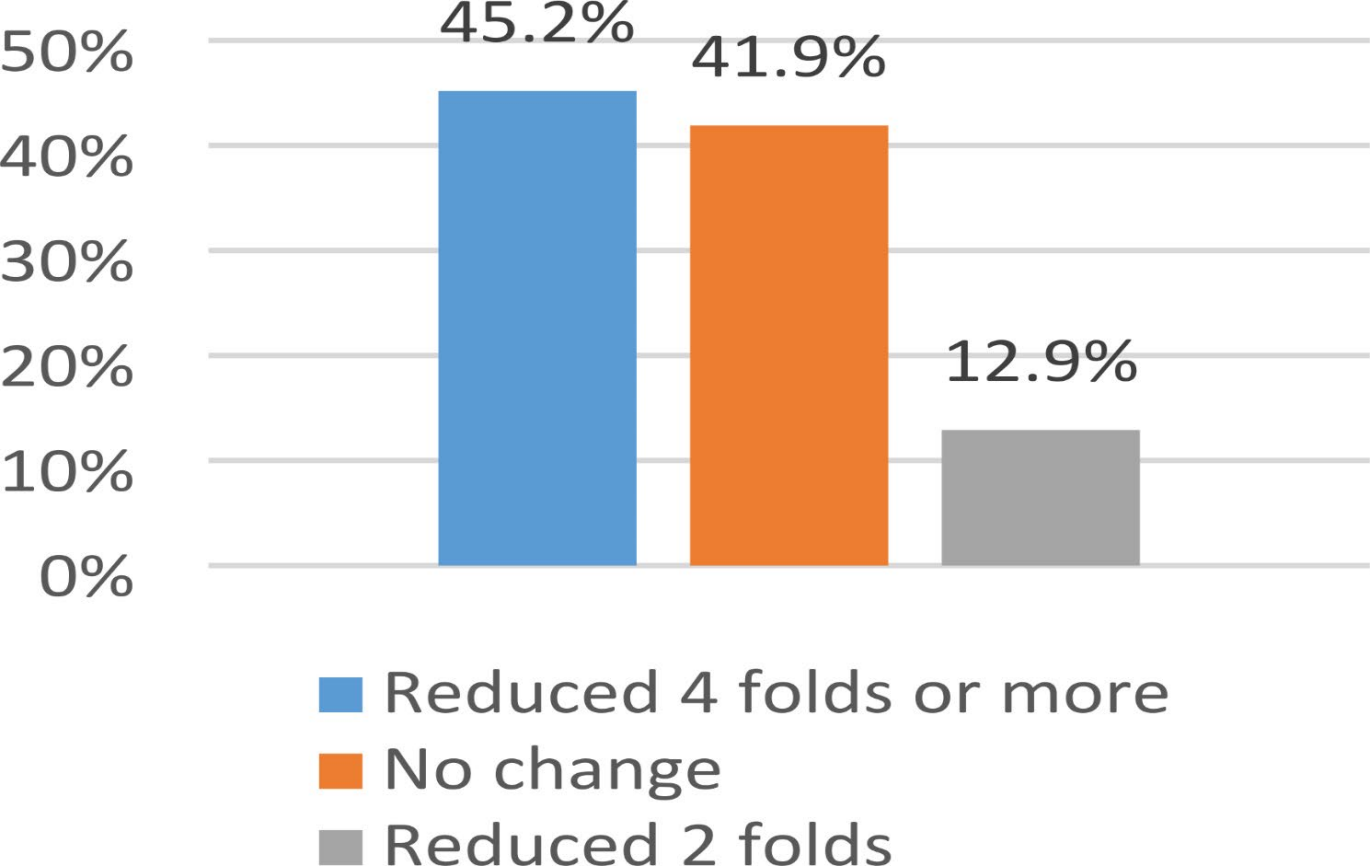
The change of MIC of tetracycline after treatment by the efflux pump inhibitor carbonylcyanide3-chlorophenylhydrazone (CCCP)

Minimal inhibitory concentration (MIC) was done using the agar dilution method, which showed 67.7% (21 isolates) resistance to tetracycline (Fig. 4). Also, the efflux pump inhibitor carbonylcyanide3-chlorophenylhydrazone (CCCP) determined the MIC of tetracycline after treatment. 45% of the isolates (14 isolates) showed a reduction of MIC 4 folds or more, and 12.9% (4 isolates) showed a 2-fold reduction in the MIC (Table 6; Fig. 5). By comparing the tetracycline resistance before and after adding CCCP, it showed a significant difference (*p < 0.05*), as the percentage of tetracycline resistance changed from 67.7% (before CCCP treatment) to 35.5% (after CCCP treatment) (Table 7). *AdeA* and *TetB* genes were detected in 100% of both resistant (21 isolates) and sensitive isolates (10 isolates). In comparison, *AdeB* was detected in 95.2% (20 isolates) of resistant and 90% (9 isolates) of sensitive isolates and *TetA* gene was detected in 85.7% (18 isolates) of resistant and 90% (9 isolates) of sensitive isolates (Table 8).

**Table 6:** MIC of the samples before and after adding CCCP, showing the effectiveness of CCCP in reduction of the MIC

Sample No.	MIC before adding CCCP (µg/ml)	MIC after adding CCCP (µg/ml)
1	128	128
2	128	128
3	4	2
4	128	128
5	4	2
6	128	128
7	128	128
8	32	0.5
9	8	0.5
10	16	0.5
11	128	128
12	32	8
13	32	8
14	64	8
15	32	8
16	4	2
17	32	8
18	4	4
19	4	4
20	32	8
21	32	8
22	4	4
23	4	2
24	32	8
25	128	128
26	4	4
27	4	0.5
28	128	128
29	64	16
30	128	128
31	32	8
Mean ± SD Median(range)	52.52 ± 11.3 32(4-128)	44.84 ± 18.5 8(0.5–128)
P-value	0.002*	

^*^ Wilcoxon sign rank test

**Table 7:** Comparison of tetracycline resistance before and after adding CCCP

	MIC of specimens before adding CCCP (µg/ml)	MIC of specimens after adding CCCP (µg/ml)	*P*-value
Resistant	21(67.7%)	11(35.5%)	0.011*
Not resistant	10(32.3%)	20(64.5%)	

*Statistically significant as *p* < 0.05, McNemar test used

**Table 8:** Distribution of *AdeAB*, *TetA*, and *TetB* genes among *A. Baumannii* isolates regarding MIC of samples (µg/ml)

	Resistant (no.=21)	Sensitive (no.=10)	*P*-value*
*AdeA* gene*-*positive isolates (no.=31)	**21(100%)**	**10(100%)**	**--**
*TetB* gene*-*positive isolates (no.= 31)	**21(100%)**	**10(100%)**	**--**
*TetA* gene*-*positive isolates (no.=27)	**18(85.7%)**	**9(90%)**	1.00
*AdeB* gene*-*positive isolates (no.=29)	**20(95.2%)**	**9(90%)**	1.00

* Fisher exact test

## Discussion

Alarmingly, the MDR *A. baumannii* infection is spreading throughout the world. It is linked to several ailments in hospitalized patients, particularly in intensive care units. Because it is a pathogen with a high propensity to acquire and/or donate resistance genes to neighboring bacteria, it is currently on the priority lists of healthcare-associated organizations, including the World Health Organization (WHO), the National Institute of Health (NIH), and the Centers for Disease Control and Prevention (CDC). It has been identified as one of the six bacterial infections of greatest healthcare concern, ESKAPE (*Enterococcus*,* Staphylococcus*,* Klebsiella*,* Acinetobacter*,* Pseudomonas*,* and Enterobacter*) *spp*., with fatality rates in some patient populations reaching 80% [10]. Numerous virulence factors that have been found over time are thought to contribute to the pathogenesis of *A. baumannii* [3]. Physical elements like its hydrophobic surface; outer membrane structures like the outer membrane protein A (OmpA) [6]; lipopolysaccharides (LPS); and complex secretory apparatus like Type II [7] and Type VI [8] secretion systems, along with their substrates [9, 10] are among them. *A. baumannii* also possesses vital mechanisms for acquiring nutrients [11, 12]. These and other virulence factors work together to enable A. baumannii to create biofilms, colonize the host, elude the host’s defenses, and spread infection throughout the body’s organs [3, 13].

The study was carried out on 457 clinical samples, a total of 31 *A. baumannii* isolates were recovered (6.78%). The age of the patients ranged from 13 days old to 72 years old, with a percentage of (54.8%) from male patients and (45.2%) from female patients (Table 2). The MDR *A. baumannii* isolates were most recovered from the ET aspirates (48.3%) samples. The bacteria were also isolated from other clinical samples; urine (16.1%), sputum (10%), blood (10%), pleural effusion (6.2%), pus (6.2%), and wound swabs (3.2%) (Table 3). The most common sources of such isolates were from the ICUs (48.3%). The bacteria were also isolated from other hospital wards; Internal Medicine (16.2%), Surgery (9.7%), NICU (6.4%), Burn Unit (6.4%), PICU (6.4%), Urology (3.3%), and Orthopedics (3.3%) wards (Table 4).

The disparities in isolation rates across studies may be related to changes in the hospital setting, the quantity of specimens examined, and shifts in the clinical status of the patients. Studies from other hospitals in Egypt showed a low prevalence rate of *A. baumannii*. It was 20%, 16.1%, 11.4%, 10% in Benha [27], Ain Shams- Cairo [28], Menoufia [29], and Cairo [30] University Hospitals respectively. A lower prevalence rate was observed in India (3%) [31], whereas higher prevalence rates were also detected in India (24.8%) [32] and (42.9%) [33].

As demonstrated by the results of our study, in a Tertiary Care Hospital in Nepal, the majority (49.18%) were *A. baumannii* isolated from specimens related to the respiratory system, such as tracheal aspirate, bronchoalveolar lavage, and sputum. Similarly, ICU patients accounted for the greatest percentage of MDR isolates (60%), followed by surgical wards (22%) and medical wards (13%), with burn wards accounting for the lowest percentage (1%) [34]. When the study was repeated after 5 years, the results showed that the majority (47.2%) were isolated from specimens related to the respiratory system, such as tracheal aspirate, bronchoalveolar lavage, and sputum. Of all the MDR isolates, 58.3% came from male patients and 41.7% came from female patients. Similarly, ICU patients accounted for the greatest percentage of MDR isolates (49.6%), followed by surgical wards (19.9%) and medical wards (14.3%), with burn wards accounting for the lowest percentage (1.9%) [35]. In a study conducted in Hamad General Hospital, Qatar, the most commonly identified sites of *A. baumannii* infection were the respiratory tract (48.9%). Of all the MDR isolates, 76.2% came from male patients and 23.8% came from female patients. ICU patients accounted for the greatest percentage of MDR isolates (28.6%) [36].

In the present study, the *bla*_OXA−51−like_ gene was used for the biological identification of *A. baumannii*. It is evident that the *bla*_OXA−51−like_ gene is found in the great majority of *A. baumannii* isolates. Their detection could offer a quick and easy way to identify *A. baumannii* that would be more dependable than the most widely used biochemical identification method (Table 5) and easier to carry out than the current definitive methods, such as amplified rRNA gene restriction analysis if they are consistently found and unique to this species. *A. baumannii* is the most important species in terms of clinical implications, thus being able to quickly differentiate it from other members of the genus would be quite beneficial [37]. In our study, the 31 isolates (100%) were confirmed to be *A. baumannii* by conventional PCR detection of the *bla*_OXA−51−like_ gene by gel electrophoresis at 353 bp (Fig. 1).

Previous studies also used the *bla*_OXA−51−like_ gene for the detection of *A. baumannii*. It was proved that the *bla*_OXA−51−like_ gene was the most prevalent gene carried by *A. baumannii* isolates. Previous studies in EGYPT demonstrated the provenance of the *bla*_OXA−51−like_ gene in (95%) of Ain Sham-Cario University Hospitals isolates [28], (96%) of Assiut University Hospitals isolates [38], and (100%) of Kasr Al-Aini -Cairo Hospital [39]. Several studies conducted in the Middle East; in Yemen [40], Kuwait [41], Saudi Arabia [42], and Qatar [43], also confirmed the prevenance of the *bla*_OXA−51−like_ gene in *A. baumannii* isolates.

Among the first β-lactamases found to be mediated by plasmids were the OXA β-lactamases. To date, more than 220 OXA-type-β-lactameases have been found. At first, they were confined to penicillins, but some of them developed the ability to confer resistance to cephalosporins. During the 1980s, isolates of carbapenem-resistant *A. baumannii* (CRAB) were revealed to exhibit plasmid-encoded β-lactamases, including OXA-23, OXA-40, OXA-58, and OXA-51. Ultimately, it was discovered that each strain of *A. baumannii* had a chromosomally encoded OXA-51, which was accountable for its antimicrobial resistance [44]. The *bla*_OXA−23−like_, *bla*_OXA−24−like_, *bla*_OXA−51−like_, *bla*_OXA−58−like_, and more recently, *bla*_OXA−143−like_ are the five primary phylogenetic OXA-type subgroups that have been identified in CRAB [45]. According to reports, *bla*_OXA−51_ gene is inherent to *A. baumannii* and chromosomally encoded, whereas plasmids or chromosomes mediate *bla*_*OXA−23*_, *bla*_*OXA−24*_, and *bla*_*OXA−58*_ [44]. Hence, the *bla*_OXA−51−like_ gene has been utilized as a marker for *A. baumannii* because every strain of the bacteria had a chromosomally encoded OXA β-lactamase (OXA-51-like). Nonetheless, non-*A. baumannii* species plus carbapenem-susceptible *A. baumannii* have been shown to harbor plasmids encoding OXA-51 [46].

In this study, the efflux system such as AdeAB, TetA, and TetB, was detected using the traditional PCR to detect various efflux pump genes, in recognition of their important mechanistic role in antibiotic resistance involving the extrusion of antimicrobials, such as tetracycline, from cells into the external environment [7]. Our research discovered *AdeA* and *TetB* genes in 100% of isolates, whereas *AdeB* was found in 93.5% of isolates and the *TetA* gene in 87.1% of isolates (Fig. 2A-D). Our findings suggest that the efflux pumps mediated by the *TetA*, *TetB*, and *AdeAB* genes play a crucial role in increasing resistance to various antibiotics, particularly tetracyclines.

Our findings match previous studies performed in EGYPT. The *AdeA* gene, which encodes one of the proteins that make up the tripartite system of the AdeABC efflux pump, was shown to be the most prevalent (82%) in clinical *A. baumannii* strains isolated from Benha University Hospital [47]. Comparable results were reported in Alexandria University Hospital, where most isolates harbored efflux pump encoding genes, as detected in our results, *AdeA*, *AdeB*, and other genes [48].

The results of our study are consistent with the results of studies around the world. The *AdeA* and *AdeB* genes were detected in 83.9% and 90.3% respectively of *A. baumannii* isolates recovered from an Iraqi hospital [49]. In a study from Iran, the prevalence of tetracycline resistance genes among tetracycline-resistant *A. baumannii* isolates was 99.2%, 86.7%, and 10% for *AdeB*, *TetB*, and *TetA* genes respectively [50]. In another study in Iran, the prevalence of the *AdeB* gene was 100% of the isolated *A. baumannii* [51]. In *A. baumannii* isolates recovered from patients in a teaching hospital in Jordan, the *TetB* gene is the most common (82.6%), while none of the isolates had the *TetA* gene [52]. The majority of the *A. baumannii* isolates recovered from Malaysian hospitals carried the *AdeA* gene (62.7%) [53]. Nemec et *al* in France reported the prevalence of the *AdeA* gene (81.9%) [54].

Different mechanisms of resistance to numerous antibiotic classes are displayed by *A. baumannii*. Efflux pumps are one of the resistance mechanisms found in *A. baumannii*. Antibiotics and other chemicals seep out of the bacteria due to these pumps, lowering the number of drugs in the bacterial cells [7]. The tetracycline-specific efflux pump system in *A. baumannii* comprises the collaborative function of TetA and TetB, which belong to the MFS superfamily of efflux pumps, alongside AdeABC, which is part of the RND superfamily of efflux pumps [9]. Several “Tet efflux pumps” that lead to resistance to tetracycline have been acquired by clinical isolates of *A. baumannii*. The TetA and TetB are the most prevalent, with “TetA efflux” conferring resistance to tetracycline but not to minocycline or doxycycline and “TetB efflux” conferring resistance to tetracycline and minocycline but not to tigecycline. It has also been found that “TetA efflux” acts synergically with the “AdeABC efflux” pumps to provide *A. baumannii* antimicrobial resistance [6]. The AdeABC efflux pump consists of three proteins; “AdeA (inner membrane fusion), AdeB (multidrug transmembrane transporter), and AdeC (outer membrane)”, which are chromosomally regulated by “adeS (sensor kinase) and adeR (response regulator)”. The two proteins work together to regulate efflux pump gene expression in response to environmental stimuli [47].

In the present study, all *A. baumannii* isolates were MDR showing resistance to three or more classes of antibiotics **(**Fig. 3**)**. Bacterial isolates that are resistant to at least one agent in at least three classes of antibiotics are classified as multidrug-resistant (MDR), while isolates that are responsive to just one or two antimicrobial classes are classified as extensively drug-resistant (XDR) [21]. Drug-resistant *A. baumannii* infections are significantly harder to treat due to the development of drug resistance brought on by its distinct physiological traits. Multiple antibiotic resistance index (MAR) was determined using the formula “MAR = x/y, where x was the number of antibiotics to which test isolate displayed resistance and y was the total number of antibiotics to which the test organism has been tested” [28].

Previous studies from other hospitals in Egypt confirmed a high prevalence of MDR *A. baumannii* isolates. It was 100%, 100%, and 61.4% in Benha [27], Ain Shams- Cairo [28], and Menoufia [29] University Hospitals respectively. International Medical Center (IMC), Kobry El-Kobba, and Al-Ganzouri Specialized Hospital- Cairo, MDR *A. baumannii* isolates represented 88.8% of the total *A. baumannii* isolates [37]. In a Tertiary Care Hospital in Nepal, 91% of *A. baumannii* isolates were MDR [35]. In Hamad General Hospital, Qatar, 95% of A. baumannii were MDR [36].

The high prevalence rates of MDR *A. baumannii* isolates worldwide, result from the resistance gene’s ability to transfer and appear anywhere in the hospital setting. *A. baumannii* has several defense mechanisms that help it avoid being affected by antimicrobial drugs. Among these processes are efflux pumps, which use efflux mechanisms to remove antibiotics from the cell, and changes made to outer-membrane proteins (OMPs) to lessen porin permeability. Furthermore, A. baumannii produces metallo-β-lactamase enzymes, including “Seoul imipenemase (SIM), New Delhi metallo β-lactamase (NDM), Verona integron-mediated metallo-β-lactamase (VIM), and imipenemase (IMP)”, By which antibiotics can be hydrolyzed such as carbapenems, penicillins, cephalosporins, and monobactams [55].

The *A. baumannii*-resistant strains continue to appear and spread, and there aren’t many effective treatment choices. For this reason, alternative antibiotics have been studied for use in clinical settings. Tetracyclines, such as doxycycline and minocycline, have demonstrated encouraging microbiological and clinical success in the treatment of *A. baumannii* infections as monotherapy or in combination with other therapies. It has been found that tetracyclines can be successfully used in conjunction with other antibiotics to treat 87.5% of bloodstream infections and 71.9% of respiratory infections [6]. Unfortunately, tetracyclines-resistant *A. baumannii* are evolving as a result of the efflux pump [7, 9]. The current study demonstrated 71% resistance to tetracycline and 58% to doxycycline.

Previous studies confirmed the spread of tetracyclines-resistant *A. baumannii*. The frequency of resistance in the studied isolates was 31.7% against tetracycline from three various hospitals in Erbil City -Iraq [56], 43.5%, and 39% against tetracycline and doxycycline respectively in the hospital of Hillah City- Iraq [57], 60%, and 50% against tetracycline and doxycycline respectively in a tertiary care hospital in North India [58], 65.4% against tetracycline in three tertiary hospitals in Jordan [59], 62% against tetracycline in Leiden University Medical Center in Netherland [54], 91.6% against tetracycline in three hospitals in Tehran City-Iran [60], and 96.3% against tetracycline in Mashhad City-Iran [61].

The high prevalence of antibiotic resistance to commonly given antibiotics in the current study worries the healthcare system since it significantly influences patient care and represents one of the main problems in the treatment of life-threatening infections caused by *A. baumannii*. This may be because these antimicrobial agents are easily accessible, frequently used outside hospitals, and readily available over the counter for self-medication [60].

In our study, the presence of active efflux systems was investigated in tetracycline-resistant *A. baumannii* isolates. The minimal inhibitory concentration (MIC) of tetracycline was assayed using “the agar dilution method before and after treatment” with the efflux pump inhibitor with carbonylcyanide 3-chlorophenylhydrazone (CCCP). 45% of the isolates showed a reduction of MIC 4-fold or more, and 12.9% showed a 2-fold reduction in the MIC (Table 6; Fig. 5). The difference in MIC results before and after treatment with CCCP showed a significant difference (*p < 0.05*), as the percentage of tetracycline resistance changed from 67.7% (before CCCP treatment) to 35.5% (after CCCP treatment) (Table 7). The efflux pump genes *AdeA*, *TetB*, AdeB, and *TetA* were detected in 100%, 100%, 95.2%, and 85.7% of tetracycline-resistant *A. baumannii* isolates (Table 8).

Several previous studies showed similar findings. Mohammed and Arif discovered that adding CCCP reduced the MIC by at least 4-fold in 22.5% of all *A. baumannii* isolates [49]. Beheshti et al.. also reported that the efflux pump inhibitor reduced the MIC 4-16-fold in the presence of the efflux pump inhibitor in 91.48% of tetracycline-resistant *A. baumannii* isolates [6]. Ardehali et al.. reported similar results, indicating that CCCP reduced the MIC of 51.25% of tigecycline-resistant *A. baumannii* isolates by 2-4-fold [10]. Ranjbar et al. reported that CCCP reduced the MIC by 4- to 8-fold for 87%, 90%, and 62% of tetracycline-, doxycycline- and minocycline-resistant *A. baumannii* isolates [62]. That was consistent with our findings, as active efflux pumps may be implicated in the higher rate of tetracycline resistance in *A. baumannii*. The significant reduction in MIC levels upon efflux pump inhibition suggests that efflux pump inhibitors could be a viable adjunctive therapy to restore tetracycline efficacy in MDR *A. baumannii* infections. Future clinical trials are warranted to explore this potential.

Consistent with these findings, a previous study confirmed the prevalence of *TetB* and *TetA* genes in at least 50% and 14%-46% of tetracycline-resistant *A. baumannii* isolates [63]. Another study showed a high prevalence (61.7%) of *TetB* but not of *TetA* gene in tetracycline-resistant isolates [6]. Comparably, *TetB* was found in a notable proportion of *A. baumannii* isolates (100% and 95%, respectively) in two separate studies conducted in Iran by Meshkat et al. [64] and Mosavat et al. [65]; surprisingly, TetA was not detected in any of the isolates of such studies.

Efflux pump inhibitors such as CCCP are still in preclinical stages and face regulatory approval challenges due to limited data on their safety in humans; future work involves validating these inhibitors in vivo and assessing their compatibility with antibiotics as adjunctive therapies; rigorous testing and demonstration of safety and efficacy are necessary before considering clinical applications [66]. Efflux pump inhibitors like CCCP are known to disrupt proton gradients, which raises concerns about their toxicity and potential off-target effects. Although it effectively lowers the MIC of some antibiotics, its application is limited due to its nonspecific mechanism and potential for cellular toxicity, studies highlight the need for alternative, safer efflux pump inhibitors with similar efficacy but better safety profiles [67]. Recent studies have shown that CCCP successfully lowers antibiotic resistance in *A. baumannii* by considerably reducing the MIC of drugs such as cefepime and imipenem. This emphasizes its potential for use in combination therapy to prevent multidrug resistance. However, more research is needed to better understand its specific use and mechanisms of action [11].

Our study recommends strict adherence to infection control procedures to stop the spread and emergence of MDR *Acinetobacter spp.* isolates, particularly in hospitals. Tetracyclines should be used with active efflux pump inhibitors to treat MDR *Acinetobacter spp.* infections. We also advise hospitals to use tetracycline medications judiciously and implement antibiotic stewardship initiatives. We will expand our study in further research to encompass resistance patterns in a variety of patient demographics, including age, gender, and underlying diseases; specimen types, such as blood, sputum, and urine; and hospital wards, such as intensive care unit (ICU) versus non-ICU. This strategy will evaluate the effects of clinical and demographic variables on efflux pump expression and resistance mechanisms using a larger, multicenter sample pool. To provide a more thorough knowledge of multidrug resistance in *A. baumannii*, we will also investigate how efflux pumps interact with other resistance mechanisms, such as enzymatic degradation and porin changes. Our findings will become more clinically relevant and applicable as a result of these efforts.

Our study still has some limitations, (1) the small sample size of the study; (2) including the single-center design where samples were collected from Suez Canal University Hospitals in Ismailia, Egypt only; (3): the focus on a specific subset of resistance mechanisms. Further broader, multicenter studies are needed to identify other types of efflux pumps and other resistance mechanisms and assess their distribution and role in tetracycline-resistant *A. baumannii* isolates emergence and spread.

## Conclusion

This study successfully identified the distribution of *AdeAB*, *TetA*, and *TetB* genes among MDR *A. baumannii* isolates from Suez Canal University Hospitals, highlighting the significant role of efflux pumps in mediating tetracycline resistance. We also found that CCCP, as an efflux pump inhibitor, can reduce tetracycline’s MIC effectively suggesting that efflux pump inhibitors could be a viable adjunctive therapy to restore tetracycline efficacy.

## Data Availability

All relevant data are included in this published article.

## Declarations

### Clinical trials registry

Pan African Clinical Trials Registry.

Trial No.: PACTR202410825114244.

Trial URL: https://pactr.samrc.ac.za/TrialDisplay.aspx?TrialID=31981.
